# HIV testing and burden of HIV infection in black cancer patients in Johannesburg, South Africa: a cross-sectional study

**DOI:** 10.1186/s12885-015-1171-7

**Published:** 2015-03-18

**Authors:** Mazvita Sengayi, Chantal Babb, Matthias Egger, Margaret I Urban

**Affiliations:** NHLS/MRC Cancer Epidemiology Research Group, National Cancer Registry, National Health Laboratory Service, Johannesburg, South Africa; Graduate School for Cellular and Biomedical Sciences, University of Bern, Bern, Switzerland; School of Pathology, Faculty of Health Sciences, University of the Witwatersrand, Johannesburg, South Africa; Institute of Social and Preventive Medicine (ISPM), University of Bern, Bern, Switzerland; Centre for Infectious Disease Epidemiology and Research (CIDER), School of Public Health and Family Medicine, University of Cape Town, Cape Town, South Africa

**Keywords:** HIV testing, Cancer, South Africa

## Abstract

**Background:**

HIV infection is a known risk factor for cancer but little is known about HIV testing patterns and the burden of HIV infection in cancer patients. We did a cross-sectional analysis to identify predictors of prior HIV testing and to quantify the burden of HIV in black cancer patients in Johannesburg, South Africa.

**Methods:**

The Johannesburg Cancer Case–control Study (JCCCS) recruits newly-diagnosed black cancer patients attending public referral hospitals for oncology and radiation therapy in Johannesburg . All adult cancer patients enrolled into the JCCCS from November 2004 to December 2009 and interviewed on previous HIV testing were included in the analysis. Patients were independently tested for HIV-1 using a single ELISA test . The prevalence of prior HIV testing, of HIV infection and of undiagnosed HIV infection was calculated. Multivariate logistic regression models were fitted to identify factors associated with prior HIV testing.

**Results:**

A total of 5436 cancer patients were tested for HIV of whom 1833[33.7% (95% CI=32.5-35.0)] were HIV-positive. Three-quarters of patients (4092 patients) had ever been tested for HIV. The total prevalence of undiagnosed HIV infection was 11.5% (10.7-12.4) with 34% (32.0–36.3) of the 1833 patients who tested HIV-positive unaware of their infection. Men >49 years [OR 0.49(0.39–0.63)] and those residing in rural areas [OR 0.61(0.39–0.97)] were less likely to have been previously tested for HIV. Men with at least a secondary education [OR 1.79(1.11–2.90)] and those interviewed in recent years [OR 4.13(2.62 – 6.52)] were likely to have prior testing. Women >49 years [OR 0.33(0.27–0.41)] were less likely to have been previously tested for HIV. In women, having children <5 years [OR 2.59(2.04–3.29)], hormonal contraceptive use [OR 1.33(1.09–1.62)], having at least a secondary education [OR:2.08(1.45–2.97)] and recent year of interview [OR 6.04(4.45–8.2)] were independently associated with previous HIV testing.

**Conclusions:**

In a study of newly diagnosed black cancer patients in Johannesburg, over a third of HIV-positive patients were unaware of their HIV status. In South Africa black cancer patients should be targeted for opt-out HIV testing.

**Electronic supplementary material:**

The online version of this article (doi:10.1186/s12885-015-1171-7) contains supplementary material, which is available to authorized users.

## Background

HIV-1 infection was classified as a human carcinogen by the International Agency for Research on Cancer (IARC) in 1996 [1]. South Africa has the largest HIV burden worldwide with an estimated 6.4 million people living with HIV in mid-2012 [2]. Black Africans are disproportionately affected by the epidemic compared to other population groups. According to the South African 2012 HIV prevalence survey, prevalence in the reproductive age group (15–49 years) was 22.7%, 0.6%, 4.6% and 1.0% in blacks, whites, coloureds (mixed race) and Indians/Asians respectively [2]. In order to understand the burden and spectrum of HIV-related cancers, it is essential to identify people with HIV and cancer co-morbidity. Strategies to identify patients with both HIV infection and cancer include screening for cancers at HIV clinics [3] and testing cancer patients for HIV [4,5].

The 2010 South African HIV counselling and testing guidelines recommend provider-initiated HIV testing and counselling (PITC) for all patients attending healthcare facilities [6]. PITC is defined as “HIV testing and counselling that is initiated and offered by health-care providers to all clients attending health-care facilities as a standard component of care” [6]. The World Health Organization recommends an ‘opt-out’ approach to PITC in generalised HIV epidemics (i.e. where antenatal HIV prevalence exceeds 1%), which not only obliges health care workers to offer HIV testing to every patient, but also incorporates the informed right of the patient to decline the recommendation of an HIV test [7]. In this paper, PITC and opt-out HIV testing will be used synonymously.

PITC has been shown to increase HIV testing almost three-fold in primary care clinics in Gauteng province, South Africa, compared to referral to on-site voluntary counselling and testing services [8]. Challenges to widespread opt-out HIV testing include overburdening a strained healthcare system, ensuring confidentiality in shared consulting rooms, on-going staff training and preventing test kit stock-outs [8]. Therefore there is still a role for targeted PITC among high risk groups, including in antenatal and post-natal clinics, tuberculosis treatment facilities, family planning clinics, sexually transmitted infections clinics and post-exposure prophylaxis centres [6]. Indeed, a recent systematic review of studies in antenatal care concluded that the adoption of PITC can greatly increase testing uptake [9].

HIV testing patterns in the general South African population have been studied: a representative survey in 2005 found that being female, employed, aged 25–34 years, having a higher education and residing in an urban area were all associated with greater knowledge of HIV status [10]. Women have consistently been found to be more likely to know their HIV status than men [2,10-15], even after accounting for pregnancy-related HIV testing [11]. The youth [14], older women [15] and older men [11,12] are less likely to be tested; the majority of older men who test for HIV generally do so when there is a medical indication [11]. Little is known about HIV testing patterns and the burden of HIV infection in cancer patients [4]. The aim of this study was to identify factors associated with HIV testing and to quantify the burden of HIV in newly diagnosed black cancer patients in Johannesburg, Gauteng province, South Africa.

## Methods

### Ethics statement

The study was approved by the University of the Witwatersrand Human Research Ethics Committee (Medical).

### Study setting and design

Since 1995, the Johannesburg Cancer Case–control Study (JCCCS) has recruited newly diagnosed self-defined black cancer patients attending public referral hospitals (Chris Hani Baragwanath, Hillbrow and Charlotte Maxeke Johannesburg Academic Hospital) for oncology and radiation therapy in the greater Johannesburg area [16]. To date, over 20 000 black cancer patients have been interviewed, of whom the majority (over 90%) have donated blood specimens for evaluation of infectious and genetic risk factors for cancer and their interactions with socio-demographic and environmental factors collected via questionnaire. At the time of the current analysis, the JCCCS patient recruitment was confined to only Charlotte Maxeke Johannesburg Academic Hospital (CMJAH) (formerly Johannesburg General Hospital).

### Eligibility criteria

All patients with confirmed cancer, aged ≥ 18 years, who were enrolled into the JCCCS at CMJAH from November 2004 to December 2009 and who were interviewed on previous HIV testing, were included in this study.

### Procedures and definitions

Trained nurse counsellors used a standard questionnaire to interview cancer patients in their preferred language (usually Zulu or Sotho). All patients gave written or witnessed verbal informed consent prior to being interviewed and having blood drawn. Patients were asked questions on place of birth, rural or urban residence, schooling, reproductive history, use of hormonal contraception, number of lifetime sexual partners, and alcohol and tobacco use. From November 2004, in addition to the standard questionnaire, all patients aged 55 or less were asked about previous HIV testing. After November 2006, all recruited patients were interviewed on prior HIV testing. Patients were asked the following questions to explore their HIV testing patterns: Have you ever been tested for HIV? Were you tested: before this illness/at time of current illness only? Were you given the test results? Are you willing to disclose your status? If yes, are you HIV positive or negative?

Nurse counsellors took blood samples from consenting patients at the time of interview, before patients had received any cancer treatment. Blood samples were collected in 10 ml red-top plain vacutainers. Serum separation was done by standard centrifugation, and serum specimens were frozen at −20 to −30°C before batching for HIV testing. Specimens were tested for HIV-1 using a single Vironostika (HIV Uniform II plus O) micro enzyme-linked immunosorbent assay (ELISA) test. Specimens with inconclusive ELISA tests were classified as HIV negative. All tests were done at the Serology Laboratory, Centre for HIV and STIs, National Institute for Communicable Diseases, Johannesburg.

Prior HIV testing was defined as self-reported history of HIV testing before the current cancer illness. Awareness of HIV status was defined as self-reported HIV status consistent with the result of the HIV-1 ELISA test. Cancers were classified according to the International Classification of Diseases for Oncology Third Edition (ICD-O-3) [17]. We classified cervical cancer (ICD-O topography code C53), Kaposi sarcoma (ICD-O morphology code M91403) and non-Hodgkin lymphoma (ICD-O topography codes C82-83 or ICD-O morphology code M9590 – 9595, M9670 – 9717 and M9820 - 9837) as AIDS-defining cancers [18]. The majority (98.4%) of patients included in the analysis had verification of cancer diagnosis by histology, haematology or cytology. Coding of cancers was done by an experienced coder working for the JCCCS and quality controlled by one of the authors (MIU) who has extensive coding expertise; assistance from oncologists, histopathologists and/or cytologists was sought where needed.

### Statistical analyses

We used means and proportions to describe the characteristics of newly diagnosed cancer patients. We determined the ten most frequent cancers in men and in women by HIV status and calculated the prevalence of prior HIV testing, of HIV infection and of undiagnosed HIV, with exact binomial 95% confidence intervals (CI). We compared age-specific HIV prevalence in male and female black cancer patients with HIV prevalence in black men and women in the South African general population, as reported in the 2008 national HIV prevalence survey [19]. We also calculated prevalence of HIV and undiagnosed HIV by cancer type (see Additional file 1: Table S6).

We fitted logistic regression models to identify factors associated with prior HIV testing in black cancer patients. Separate models were calculated for men and women. The following variables were entered into the models: age (≤49 years, >49 years), place of residence (rural vs. urban), marital status (married, single, widowed, divorced), year of interview, highest level of education achieved (none, primary and secondary/tertiary), alcohol use (non-drinkers, moderate drinkers and heavy/binge drinkers), smoking (non-smokers, ex-smokers, current light smokers and current heavy smokers), lifetime number of sexual partners (0–1, 2–5 and 6 or more), having children under the age of five, cancer type (AIDS defining or other cancers), hormonal contraceptive use (ever, never) and interviewer. We had three nurse interviewers: interviewer 1, the most senior interviewer, was with the study during the entire period; interviewer 3 replaced interviewer 2 in September 2009. We classified alcohol use as follows: non-drinkers (<1 drink per week), moderate drinkers (1–7 drinks/week for women, 1–14 drinks/week for men) and heavy/binge drinkers (8 or more drinks/week or 4 or more drinks in a single occasion for women, 15 or more drinks/week or 5 or more drinks in a single occasion for men) [20]. We considered that some patients may have quit smoking due to their illness; hence those who stopped smoking more than 5 years prior to the date of interview were classified as ex-smokers while those who smoked within 5 years of the date of interview were classified as current smokers. Current smokers were further classified into ‘light’ (1 – 14 g/day) and ‘heavy’ (15 g/day or more) current smokers, assuming weights of 1 g per cigarette or hand rolled cigarette or pipe [21].

We added variables to multivariate models sequentially, starting with variables with the smallest p value in univariate analysis. Likelihood ratio tests were performed to determine which variables were kept in the models. The final model for men was adjusted for age, place of residence, highest level of education achieved, year of interview and interviewer. The final model for women was adjusted for age, highest level of education achieved, year of interview, having children under the age of five, hormonal contraceptive use and interviewer. Stata software (version 13, Stata Corporation, College Station, Texas, USA) was used for all analyses.

## Results

### Characteristics of cancer patients

**Figure 1:**
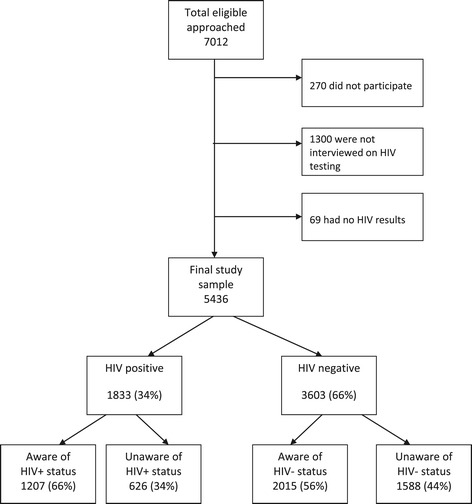
**Flowchart of cancer patient recruitment (2004 – 2009).**

Out of the 7012 eligible patients who were approached, 270 did not participate, 1300 were not interviewed on HIV testing and 69 did not have HIV screening test results (Figure 1). Reasons for non-participation were: too sick/in pain 105 (39%), unable to communicate 56 (21%), refused 44 (16%), left before interview 33 (12%), impaired cognitive function 28 (10%) and other 4 (2%). The remaining study sample had 5436 patients with HIV test results. Two thirds of patients were female and 54% patients were 49 years old or younger (Table 1). The majority (89%) were urban dwellers and 58% were married or lived with their partner. Forty percent of patients had completed primary education or less. Fifteen percent were heavy/binge alcohol drinkers and 28% were current smokers. A quarter of all patients had had more than five sexual partners in their lifetime. Only 13% had children under the age of five. The majority of women (63%) had previously used hormonal contraceptives. Missing data were ≤2% for all variables, except for the variable “having children under 5 years”, where 9.3% of data were missing.

**Table 1:** **Characteristics of newly diagnosed black South African cancer patients (2004 – 2009)**

**Characteristics**	**N**	**%**
**Age**		
≤49 years	2954	54.3
>49 years	2482	45.7
Mean (Standard deviation)	47.8 (12.2)	
**Place of residence**		
Urban	4840	89.0
Rural	591	10.9
Missing data	5	0.1
**Gender**		
Male	1793	33.0
Female	3643	67.0
**Marital status**		
Married/Living together	3147	57.9
Single/Never married	680	12.5
Widowed	737	13.6
Separated/Divorced	863	15.9
Missing data	9	0.2
**HIV Screening test result**		
Negative	3603	66.3
Positive	1833	33.7
**Level of education**		
None	549	10.1
Primary	1641	30.2
Secondary/Tertiary	3238	59.6
Missing data	8	0.2
**Alcohol use**		
Non-drinkers	3253	59.8
Moderate drinkers	1342	24.7
Heavy/Binge drinkers	841	15.5
**Smoking**		
Non-smokers	3436	63.2
Ex-smokers	457	8.4
Current smokers (1 – 14 g/day)	1099	20.2
Current smokers (15+ g/day)	444	8.2
**Lifetime number of sexual partners**		
0-1	585	10.8
2-5	3401	62.6
6+	1343	24.7
Missing data	107	2.0
**Having children under 5 years**		
No	4233	77.9
Yes	700	12.9
Missing data	503	9.3
**Cancer type**		
AIDS-defining	1923	35.4
Other cancers	3513	64.6
**Hormonal contraceptive use (Women only)**		
Never	1329	36.5
Ever	2299	63.1
Missing data	15	0.4
**Interviewer**		
Interviewer 1	3161	58.2
Interviewer 2	2074	38.1
Interviewer 3	201	3.7

### Cancers in men and women by HIV status

The five most common cancers in HIV-positive men were Kaposi sarcoma (KS), non-Hodgkin lymphoma (NHL), oro-pharyngeal, lung and naso-laryngeal cancers, totalling 75% of all cancers (Table 2). In HIV-negative men, the top five cancers were oro-pharyngeal, lung, oesophageal, naso-laryngeal and stomach cancers, totalling 54% of all cancers. For HIV-positive women, the five most common cancers were cervical, breast, KS, NHL and oro-pharyngeal cancer, totalling 84% of all cancers. In HIV-negative women, the top five cancers were breast, cervical, oesophageal, ovarian and uterine cancer, totalling 77% of all cancers.

**Table 2:** **Ten most frequent cancers in black South African men and women by HIV status (2004 – 2009)**

**HIV positive men (n = 596)**		**HIV negative men (n = 1197)**	
Kaposi sarcoma	260 (43.6)	Lip, oral cavity and pharynx	216 (18.1)
NHL	94 (15.8)	Lung	167 (14.0)
Diffuse large B cell lymphoma	54 (9.1)		
Burkitt’s lymphoma	6 (1.0)		
Other NHL	34 (5.7)		
Lip, oral cavity and pharynx	38 (6.4)	Oesophagus	125 (10.4)
Lung	34 (5.7)	Nasal cavity and larynx	82 (6.9)
Nasal cavity and larynx	21 (3.5)	Stomach	54 (4.5)
Oesophagus	19 (3.2)	Colon	45 (3.8)
Hodgkin Lymphoma	15 (2.5)	Anorectal	45 (3.8)
Liver	11 (1.9)	Liver	45 (3.8)
Stomach	9 (1.5)	Pancreas	39 (3.3)
Skin (non-melanoma, non-SCC*)	7 (1.2)	Prostate	39 (3.3)
**HIV positive women (n = 1237)**		**HIV negative women (n = 2406)**	
Cervix	448 (36.2)	Breast	874 (36.3)
Breast	241 (19.5)	Cervix	718 (29.8)
Kaposi sarcoma	226 (18.3)	Oesophagus	93 (3.9)
NHL	95 (7.7)	Ovary	89 (3.7)
Diffuse large B cell lymphoma	61 (4.9)		
Burkitt’s lymphoma	9 (0.7)		
Other NHL	25 (2.0)		
Lip, oral cavity and pharynx	26 (2.1)	Uterus	84 (3.5)
Vulva	22 (1.8)	Colon	54 (2.2)
Uterus	17 (1.4)	Lip, oral cavity and pharynx	51 (2.1)
Conjunctiva, Eye	17 (1.4)	Lung	48 (2.0)
Lung	15 (1.2)	Anorectal	39 (1.6)
Oesophagus	14 (1.1)	NHL	30 (1.3)
		Diffuse large B cell lymphoma	10 (0.4)
		Burkitt’s lymphoma	2 (0.1)
		Other NHL	18 (0.8)

Numbers (%) are shown.

*non-squamous cell carcinoma.

### HIV testing and prevalence in cancer patients

Among patients with available results, 1833 had a positive ELISA test, for a prevalence of HIV infection of 33.7% (95% CI 32.5-35.0). Out of the 1833 cancer patients with a positive HIV-1 test, 626 [34% (95% CI 32.0 – 36.3)] were unaware of their positive HIV status. The overall prevalence of previously undiagnosed HIV among all black cancer patients was 11.5% (95% CI 10.7-12.4): 626 patients out of 5436 patients with HIV test results. Among the 3603 patients who tested HIV-1 negative, 1588 (44%) were unaware of their HIV status.

**Figure 2:**
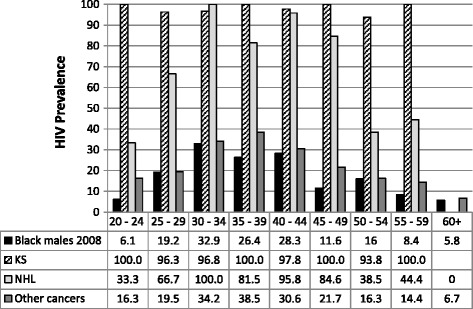
**HIV prevalence in male cancer patients (2004 – 2009).** Age-specific HIV prevalence in male cancer patients in the present study compared with age-specific HIV prevalence in black men in South Africa as reported in the 2008 National HIV Prevalence Survey.

**Figure 3:**
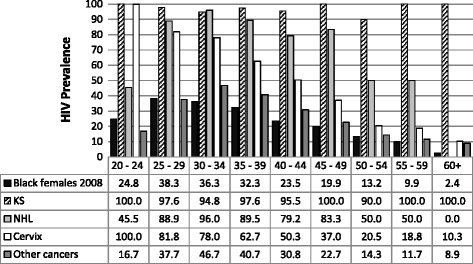
**HIV prevalence in female cancer patients (2004 – 2009).** Age-specific HIV prevalence in female cancer patients in the present study compared with age-specific HIV prevalence in black women in South Africa as reported in the 2008 National HIV Prevalence Survey.

Figure 2 and Figure 3 compare the age-specific HIV prevalence in black men and women with cancer to black men and women in the general South African population [19]. In men with KS or NHL age-specific HIV prevalence was high ranging from 96.3% to 100% for KS and 33.3% to 100% for NHL, in the age groups 20 to 60 years. In men with other cancers HIV prevalence was generally slightly higher in black cancer patients than in the black male population. HIV prevalence in black men with other cancers rose with age, peaking in the 35–39 years age group and then declining to below 10% in men 60 years or older. In women the pattern was similar to that observed in men for KS, NHL and for other cancers. The pattern differed for cervical cancer: HIV prevalence was 100% in women aged 20–24 years and steadily declined with increasing age to 10% in women aged 60 or older (Figure 3).

### Factors associated with prior HIV testing

A total of 4092 (75%) out of 5436 patients had ever been tested for HIV; of those, 1303 (32%) had been tested before their current cancer illness. The remaining 2789 (68%) had been tested for HIV at the time of their cancer illness or diagnosis. The median time elapsed since the last HIV test at time of interview was 1.9 months (IQR 1.0 – 5.6). Only 19 (<1%) out of the 4092, who had ever been tested, were unwilling to disclose the results of their previous HIV test. Factors independently associated with prior HIV testing in men included younger age, urban residence, higher level of education and more recent year of interview (Table 3). For example, men with secondary/tertiary education had a 1.79 times greater odds of a prior HIV test than men with no education. Similarly, in women, younger age, higher level of education and more recent year of interview were associated with prior testing. In addition, having children under the age of five and hormonal contraceptive use were independently associated with prior HIV testing (Table 4).

**Table 3:** **Factors associated with HIV testing in black South African men with cancer (2004 – 2009)**

**Factor**	**No previous HIV test N (%)**	**Previous HIV test N (%)**	**Crude logistic model OR (95% CI)**	**Multivariate logistic model OR (95% CI)**
**Age**				
≤49 years	641 (45.8)	238 (60.7)	1	1
>49 years	760 (54.2)	154 (39.3)	0.54 (0.43 – 0.68)	0.49 (0.39 – 0.63)
**Place of residence**				
Urban	1241 (88.6)	367 (93.6)	1	1
Rural	160 (11.4)	25 (6.4)	0.53 (0.34 – 0.82)	0.61 (0.39 – 0.97)
**Marital status**				
Married/living together	960 (68.6)	249 (63.7)	1	
Single/never married	170 (12.2)	48 (12.3)	1.09 (0.77 – 1.54)	
Widowed	99 (7.1)	36 (9.2)	1.40 (0.93 – 2.10)	
Separated/divorced	170 (12.2)	58 (14.8)	1.32 (0.95 – 1.83)	
**Year of interview**				
2004-2005	197 (14.1)	30 (7.7)	1	1
2006	235 (16.8)	38 (9.7)	1.06 (0.63 – 1.78)	1.04 (0.61 – 1.76)
2007	351 (25.1)	77 (19.6)	1.44 (0.91 – 2.27)	1.88 (1.17 – 3.02)
2008	355 (25.3)	107 (27.3)	1.98 (1.27 – 3.07)	2.44 (1.54 – 3.85)
2009	263 (18.8)	140 (35.7)	3.49 (2.26 – 5.40)	4.13 (2.62 – 6.52)
**Level of education**				
None	145 (10.4)	24 (6.1)	1	1
Primary	495 (35.4)	94 (24.0)	1.15 (0.71 – 1.86)	1.14 (0.69 – 1.89)
Secondary/tertiary	758 (54.2)	274 (69.9)	2.18 (1.39 – 3.44)	1.79 (1.11 – 2.90)
**Alcohol use**				
Non-drinkers	397 (28.3)	118 (30.1)	1	
Moderate drinkers	635 (45.3)	165 (42.1)	0.87 (0.67 – 1.14)	
Heavy/binge drinkers	369 (26.3)	109 (27.8)	0.99 (0.74 – 1.34)	
**Smoking**				
Non-smokers	318 (22.7)	100 (25.5)	1	
Ex-smokers	221 (15.8)	52 (13.3)	0.75 (0.51 – 1.09)	
Current smokers (1–14 g/day)	580 (41.4)	155 (39.5)	0.85 (0.64 – 1.13)	
Current smokers (15+ g/day)	282 (20.1)	85 (21.7)	0.96 (0.69 – 1.33)	
**Lifetime number of sexual partners**				
0-1	89 (6.5)	21 (5.5)	1	
2-5	633 (46.3)	156 (41.2)	1.04 (0.63 – 1.73)	
6+	645 (47.2)	202 (53.3)	1.33 (0.80 – 2.19)	
**Having children under 5 years**				
No	828 (79.8)	228 (75.2)	1	
Yes	210 (20.2)	75 (24.8)	1.30 (0.96 – 1.75)	
**Cancer type**				
AIDS-defining	294 (21.0)	104 (26.5)	1	
Other cancers	1107 (79.0)	288 (73.5)	0.73(0.57 – 0.95)	
**Interviewer**				
Interviewer 1	771 (55.0)	291 (74.2)	1	1
Interviewers 2 and 3	630 (45.0)	101 (25.8)	0.69 (0.63 – 0.76)	0.43 (0.33 – 0.56)

**Table 4:** **Factors associated with HIV testing in black South African women with cancer**

**Factor**	**No previous HIV test N (%)**	**Previous HIV test N (%)**	**Crude logistic model OR (95% CI)**	**Multivariate logistic model OR (95% CI)**
**Age**				
≤49 years	1368 (50.1)	707 (77.6)	1	1
>49 years	1364 (49.9)	204 (22.4)	0.29 (0.24 – 0.34)	0.33 (0.27 – 0.41)
**Place of residence**				
Urban	2392 (87.7)	840 (92.3)	1	
Rural	336 (12.3)	70 (7.7)	0.59 (0.45 – 0.78)	
**Marital status**				
Married/living together	1503 (55.1)	435 (47.9)	1	
Single/never married	280 (10.3)	182 (20.0)	2.24 (1.81 – 2.78)	
Widowed	483 (17.7)	119 (13.1)	0.85 (0.68 – 1.07)	
Separated/divorced	462 (16.9)	173 (19.0)	1.29 (1.05 – 1.59)	
**Year of interview**				
2004-2005	497 (18.2)	77 (8.5)	1	1
2006	433 (15.8)	99 (10.9)	1.47 (1.07 – 2.04)	1.61 (1.14 – 2.28)
2007	642 (23.5)	173 (19.0)	1.74 (1.30 – 2.33)	2.91 (2.10 – 4.01)
2008	648 (23.7)	231 (25.4)	2.30 (1.73 – 3.05)	3.62 (2.65 – 4.95)
2009	512 (18.7)	331 (36.3)	4.17 (3.16 – 5.50)	6.04 (4.45 – 8.21)
**Level of education**				
None	336 (12.3)	44 (4.8)	1	1
Primary	880 (32.3)	172 (18.9)	1.49 (1.04 – 2.13)	1.26 (0.86 – 1.84)
Secondary/tertiary	1511 (55.4)	695 (76.3)	3.51 (2.53 – 4.87)	2.08 (1.45 – 2.97)
**Alcohol use**				
Non-drinkers	2071 (75.8)	667 (73.2)	1	
Moderate drinkers	401 (14.7)	141 (15.5)	1.09 (0.88 – 1.35)	
Heavy/binge drinkers	260 (9.5)	103 (11.3)	1.23 (0.96 – 1.57)	
**Smoking**				
Non-smokers	2240 (82.0)	778 (85.4)	1	
Ex-smokers	152 (5.6)	32 (3.5)	0.61 (0.41 – 1.90)	
Current smokers (1–14 g/day)	278 (10.2)	86 (9.4)	0.89 (0.69 – 1.15)	
Current smokers (15+ g/day)	62 (2.3)	15 (1.6)	0.70 (0.39 – 1.23)	
**Lifetime number of sexual partners**				
0-1	383 (14.3)	92 (10.3)	1.38 (1.08 – 1.76)	
2-5	1962 (73.0)	650 (72.5)	1.87 (1.39 – 2.52)	
6+	342 (12.7)	154 (17.2)		
**Having children under 5 years**			1	1
No	2497 (92.8)	680 (75.5)	4.18 (3.39 – 5.16)	2.59 (2.04 – 3.29)
Yes	194 (7.2)	221 (24.5)		
**Cancer type**				
AIDS-defining	1140 (41.7)	385 (42.3)	1	
Other	1592 (58.3)	526 (57.7)	0.98 (0.84 – 1.14)	
**Hormonal contraceptive use**				
Never	1105 (40.6)	224 (24.7)	1	1
Ever	1615 (59.4)	684 (75.3)	2.09 (1.76 – 2.47)	1.33 (1.09 – 1.62)
**Interviewer**				
Interviewer 1	1426 (52.2)	673 (73.9)	1	1
Interviewers 2 and 3	1306 (47.8)	238 (26.1)	0.74 (0.69 – 0.78)	0.36 (0.30 – 0.44)

## Discussion

The prevalence of HIV infection in black cancer patients diagnosed in a large tertiary academic hospital in Johannesburg, South Africa was 34%, demonstrating a higher HIV prevalence in black cancer patients than in the general black population. While three-quarters of all patients had ever been tested for HIV, about a third of the infected patients were unaware of their HIV infection. Overall more than 10% of all newly diagnosed black cancer patients had a previously unknown HIV infection. The HIV prevalence in this population was substantially higher than the national South African adult HIV prevalence of 18% [19] but comparable with other high risk groups. For example, the National Antenatal Sentinel HIV and Syphilis Prevalence survey showed that 29% of pregnant women were HIV positive in 2009 [22]. HIV prevalence in cancer patients is expected to be high since HIV is a well-established risk factor for AIDS-defining cancers, but the prevalence was also higher in patients with cancers that are not classified as AIDS-defining. Overall HIV prevalence in cancer patients in a previous JCCCS publication covering the period March 1995 – June 2004, was found to be 10% [16]. The current analysis, covering the period November 2004 - December 2009 found a much higher prevalence of 34%. The difference in the HIV sero-positivity reflects the different stages of the South African HIV epidemic in the two time periods. The much lower HIV prevalence in 1990s might have affected overall HIV prevalence in previous JCCCS publications exemplified by an antenatal HIV prevalence of 10.4% in 1995 which steadily rose to 29.5% in 2004, plateauing around this level in the years 2004 to 2012 [23].

We found that younger age, higher level of education and more recent year of interview were associated with having been tested for HIV both in men and in women. Older men and women were less likely to have been tested; this is consistent with a study of age and gender differences in HIV testing uptake done in South Africa’s Mpumalanga province [11]. In Mpumalanga the peak age-group for HIV testing was 20–39 years for both men and women; older men and women were less likely to get tested and a significant proportion of older people who did test did so only after medical referral [11]. Black cancer patients interviewed in more recent years had a higher odds of prior HIV testing and, similarly, there was an increase in HIV testing in the Mpumalanga study from 2002 to 2006 [11]. Greater availability of HIV testing facilities and of antiretroviral treatment could partially explain improved testing coverage in recent years. As expected, higher level of education was associated with greater prevalence of prior HIV testing in both men and women [14,24]. Indeed, the determinants of HIV testing in black men and women with cancer are similar to those seen in the general South African population [10,11,14,15].

Women having children under the age of five or using hormonal contraceptives were also more likely to have been tested for HIV. Younger women who use hormonal contraception and who have small children would have attended health facilities where they will have been offered HIV testing [15]. Routine opt-out antenatal HIV testing in pregnant women has been part of the prevention of mother-to-child transmission of HIV (PMTCT) programme since 2001 [25], which probably explains the higher HIV testing prevalence in women with young children. This finding underscores the potential of implementation of PITC when patients with cancer and HIV co-morbidity can be identified thereby allowing for appropriate treatment and referral for HIV care.

Men and women with KS had a persistently high HIV prevalence across the age-groups, whereas for cervical cancer the prevalence was highest in young women and declined steadily with increasing age. This is consistent with the young age at cervical cancer diagnosis as previously described in HIV positive women in South Africa [26], and supports the hypothesis that HIV works as a cofactor which shortens the pre-invasive stage in cervical carcinogenesis [26]. The leading cancers in men and women differed by HIV status, with AIDS-defining cancers accounting for 59% and 62% of all cancers in HIV positive men and women respectively. The non-AIDS defining cancers Hodgkin lymphoma and skin (non-melanoma, non-squamous cell carcinoma) in men, and vulval and conjunctival cancers in women emerged in the top ten. The association of these non-AIDS defining cancers with HIV has been described before in the JCCCS [16] and in other African settings [27-30]. Anal cancer is the most common non-AIDS defining cancer in HIV positive men in Europe and the United States [31]. The conspicuous absence of anorectal cancers in the top ten cancers in HIV positive black men in our study can be explained by the predominantly heterosexual transmission of HIV in South Africa [32]. Conjunctival cancer was detected among the top ten cancers in HIV positive female black cancer patients but was not in the top ten cancers in HIV positive men. This might reflect differences in health seeking behaviours between men and women, hindering diagnosis in men. Also, it might possibly reflect an HIV-related shift from the male predominance of conjunctival cancer to a female predominance as previously observed in a study of conjunctival cancer in Zimbabwe, where 70% of conjunctival cancers were in women [33].

The overall undiagnosed HIV prevalence of 11.5% is comparable to other studies done in the general population; the prevalence of previously undiagnosed HIV was 10.3% in a population-based sero-survey done in Cape Town [13]. In the current study, over a third of HIV positive cancer patients were unaware of their HIV status. This is concerning and has implications for management of cancer patients. Undiagnosed HIV infection could potentially worsen treatment outcomes for cancer patients, who might have untreated HIV-related immunodeficiency in addition to coping with cancer-specific chemotherapy, radiation therapy or surgery. Further research is required to understand why newly diagnosed cancer patients are not routinely tested for HIV or are unaware of their HIV result.

Our analysis has some limitations. In the first 2 years (November 2004 – November 2006) of introduction of the HIV section of the questionnaire, it was only used for patients aged 55 or less; thereafter it was used for all patients. Patients not interviewed on HIV testing were therefore older, and had a lower HIV prevalence than those interviewed (see Additional file 1: Table S5). This might have inflated overall HIV prevalence in the study. Furthermore, there was potential for recall bias and differential reporting of previous HIV testing in patients who had and had not previously tested positive for HIV. Although interviews were conducted by experienced nurse counsellors there was possible social desirability bias, as patients might want to give health care workers responses which cast them favourably. Self-reported HIV status may overestimate true lack of awareness of HIV status where patients who are aware of their HIV positive status may report unknown or HIV negative status [34]. Hence HIV stigma might have affected the high prevalence of undiagnosed HIV. The study was limited to black cancer patients who were mainly from southern Gauteng province; thus the results may not apply to other provinces in South Africa or other countries in sub-Saharan Africa. This study was conducted during the period 2004–2009; since then there has been continued ART scale-up, and in 2010, national HIV testing guidelines were published and a national HIV testing campaign was implemented [6,35]. Therefore current HIV testing patterns and HIV burden in cancer patients might differ from the findings of this study. HIV testing patterns in cancer patients in South Africa had not previously been studied. The current analysis provides a baseline picture of HIV testing patterns in cancer patients in the first five years of the roll-out of antiretroviral combination therapy in South Africa. Another strength is the large sample size, which allowed for precise estimates. The majority of patients had independent study related HIV test results and most other variables were fairly complete.

## Conclusions

HIV prevalence is higher in black cancer patients in Johannesburg than in the general black population, even among patients with cancers which are not AIDS defining. Clinicians should not miss the opportunity to offer PITC to cancer patients at the time of cancer diagnosis. The HIV testing patterns in black cancer patients reflect targeted HIV testing in the reproductive age group. More than a third of newly diagnosed black cancer patients with HIV were unaware of their HIV status. This emphasises the need for implementation of PITC not only in the general population, but also in black cancer patients in South Africa’s high HIV prevalence setting. Routine opt-out HIV testing in black cancer patients should be implemented as standard of care in South Africa.

## Additional file

Additional file 1: Table S5. Differences in characteristics of patients interviewed and not interviewed on HIV testing. **Table S6.** prevalence of HIV and undiagnosed HIV by cancer type.

## Abbreviations

AIDS: Acquired immunodeficiency syndrome
ELISA: Enzyme linked immunosorbent assay
HIV: Human immunodeficiency virus
IARC: International Agency for Research on Cancer
ICD-O-3: International Classification of Diseases for Oncology, Third Edition
JCCCS: Johannesburg Cancer Case–control Study
KS: Kaposi sarcoma
NHL: Non-Hodgkin lymphoma
PITC: Provider-initiated HIV testing and counselling
PMTCT: Prevention of mother-to-child transmission of HIV
