# Supplementary material for: HIV testing and burden of HIV infection in black cancer patients in Johannesburg, South Africa: a cross-sectional study
Source: BMC Cancer. 2015 Mar 18;15:144. doi: 10.1186/s12885-015-1171-7 (PMC4434805; doi:10.1186/s12885-015-1171-7)
Supplement: Additional file 1: Table S5. — Differences in characteristics of patients interviewed and not interviewed on HIV testing. Table S6. prevalence of HIV and undiagnosed HIV by cancer type. [file 12885_2015_1171_MOESM1_ESM.doc]

Supplementary Tables

Table 5: Differences in characteristics of patients interviewed and not interviewed on HIV testing

| Characteristics | Interviewed  N (%) | Not Interviewed  N (%) | Pearson’s  Chi-squared test  P Value |
| --- | --- | --- | --- |
| **Age**  ≤49 years  >49 years | 2954 (54.3)  2482 (45.7) | 216 (17.2)  1039 (82.8) | <0.001 |
| **Place of residence**  Urban  Rural | 4840 (89.1)  591 (10.9) | 1079 (86.0)  176 (14.0) | 0.002 |
| **Gender**  Male  Female | 1793 (33.0)  3643 (67.0) | 488 (38.8)  769 (61.2) | <0.001 |
| **Marital Status**  Married/Living together  Single/Never married  Widowed  Separated/Divorced | 680 (12.5)  3147 (58.0)  737 (13.6)  863 (15.9) | 46 (3.7)  734 (58.7)  317 (25.4)  153 (12.2) | <0.001 |
| **Year of interview**  2004-2005  2006  2007  2008  2009 | 801 (14.7)  805 (14.8)  1243 (22.9)  1341 (24.7)  1246 (22.9) | 740 (58.9)  507 (40.3)  8 (0.6)  1 (0.1)  1 (0.1) | <0.001 |
| **HIV Screening Test Result**  Negative  Positive | 3603 (66.3)  1833 (33.7) | 1083 (86.2)  174 (13.8) | <0.001 |
| **Level of Education**  None  Primary  Secondary/Tertiary | 549 (10.1)  1641 (30.2)  3238 (59.7) | 243 (19.4)  493 (39.4)  517 (41.3) | <0.001 |
| **Alcohol Use**  Non-drinkers  Moderate drinkers  Heavy/Binge drinkers | 3253 (59.8)  1342 (24.7)  841 (15.5) | 723 (57.5)  326 (25.9)  208 (16.6) | 0.313 |
| **Smoking**  Non-smokers  Ex-smokers  Current smokers (1 – 14g/day)  Current smokers (15+ g/day) | 3436 (63.2)  457 (8.4)  1099 (20.2)  444 (8.2) | 666 (53.1)  171 (13.6)  303 (24.1)  115 (9.2) | <0.001 |
| **Lifetime number of sexual partners**  0-1  2-5  6+ | 585 (10.9)  3401 (63.8)  1343 (25.2) | 214 (17.3)  727 (58.9)  294 (23.8) | <0.001 |
| **Having children under 5years**  No  Yes | 4233 (85.8)  700 (14.2) | 1093 (96.2)  43 (3.8) | <0.001 |
| **Cancer Type**  AIDS-defining  Other cancers | 1923 (35.4)  3513 (64.6) | 285 (22.7)  972 (77.3) | <0.001 |
| **Hormonal Contraceptive Use (Women Only)**  Never  Ever | 1329 (36.6)  2299 (63.4) | 445 (58.3)  319 (41.7) | <0.001 |

Table 6: Prevalence of HIV and undiagnosed HIV by cancer type

| Cancer (ICD-O-3 codes) | Total | HIV Positive | | Undiagnosed  HIV infection | |
| --- | --- | --- | --- | --- | --- |
|  | N | n | % | n | % |
| Lip, oral cavity and pharynx (C00-14) | 332 | 64 | 19.3 | 35 | 10.5 |
| Oesophagus (C15) | 251 | 33 | 13.2 | 24 | 9.6 |
| Stomach (C16) | 99 | 16 | 16.2 | 10 | 10.1 |
| Small intestine (C17) | 8 | 1 | 12.5 | 1 | 12.5 |
| Colon, recto-sigmoid junction (C18-19) | 114 | 15 | 13.2 | 10 | 8.8 |
| Anorectal (C20-21) | 97 | 13 | 13.4 | 6 | 6.2 |
| Liver (C22) | 74 | 14 | 18.9 | 4 | 5.4 |
| Gallbladder & biliary tract (C23-C24) | 37 | 4 | 10.8 | 3 | 8.1 |
| Pancreas (C25) | 63 | 8 | 12.7 | 4 | 6.4 |
| Other ill-defined digestive organs (C26) | 8 | 2 | 25 | 0 | 0.0 |
| Nasal cavity and larynx (C30,C32) | 123 | 24 | 19.5 | 15 | 12.2 |
| Lung (C33,C34) | 264 | 49 | 18.6 | 26 | 9.9 |
| Thymus (C37) | 4 | 0 | 0 | 0 | 0.0 |
| Heart, mediastinum and pleura (C38) | 15 | 1 | 6.7 | 0 | 0.0 |
| Bone, joints & cartilage (C40-41) | 43 | 6 | 13.9 | 5 | 11.6 |
| Other haematopoietic cancers (C42) | 14 | 3 | 21.4 | 1 | 7.1 |
| Other skin (C44) | 25 | 11 | 44 | 7 | 28.0 |
| Nerve (C47) | 4 | 0 | 0 | 0 | 0.0 |
| Peritoneum (C48) | 11 | 0 | 0 | 0 | 0.0 |
| Connective tissue (C49) | 54 | 10 | 18.5 | 5 | 9.3 |
| Breast (C50) | 1123 | 243 | 21.6 | 161 | 14.3 |
| Vulva (C51) | 47 | 22 | 46.8 | 6 | 12.8 |
| Vagina (C52) | 5 | 3 | 60 | 3 | 60.0 |
| Cervix (C53) | 1166 | 448 | 38.4 | 124 | 10.6 |
| Uterine (C54,C55,C57) | 101 | 17 | 16.8 | 7 | 6.9 |
| Ovary (C56) | 102 | 12 | 11.8 | 6 | 5.9 |
| Placenta (C58) | 15 | 5 | 33.3 | 4 | 26.7 |
| Penis (C60) | 11 | 5 | 45.5 | 3 | 27.3 |
| Prostate (C61) | 45 | 6 | 13.3 | 6 | 13.3 |
| Testes (C62) | 7 | 2 | 28.6 | 0 | 0.0 |
| Scrotum & other male organs (C63) | 4 | 0 | 0 | 0 | 0.0 |
| Kidney (C64) | 28 | 3 | 10.7 | 2 | 7.1 |
| Bladder & Urethra (C67-68) | 31 | 1 | 3.2 | 0 | 0.0 |
| Conjunctiva, Eye (C69) | 27 | 23 | 85.2 | 11 | 40.7 |
| Meninges (C70) | 2 | 0 | 0 | 0 | 0.0 |
| Brain (C71) | 7 | 0 | 0 | 0 | 0.0 |
| Spinal cord (C72) | 1 | 0 | 0 | 0 | 0.0 |
| Thyroid (C73) | 12 | 0 | 0 | 0 | 0.0 |
| Adrenal & other endocrine glands (C74) | 5 | 0 | 0 | 0 | 0.0 |
| Ill-defined sites (C76) | 25 | 7 | 28 | 5 | 20.0 |
| Unknown primary site (C80.9) | 77 | 20 | 26 | 9 | 11.7 |
| Kaposi's sarcoma (M9140) | 499 | 486 | 97.4 | 35 | 7.0 |
| NHL (C82-83, M9590-9595, M9670-9717 & M9820-9837)  Diffuse large B cell  Burkitt’s lymphoma  Other NHL | 258  137  18  103 | 189  115  15  59 | 73.3  83.9  83.3  57.3 | 59  33  5  21 | 22.9  24.1  27.8  20.4 |
| Hodgkin’s Lymphoma (M9650-9667) | 44 | 27 | 61.4 | 10 | 22.7 |
| Myeloma & plasma cell tumours (M9731–9734) | 59 | 9 | 15.3 | 8 | 13.6 |
| Myeloid leukaemias (M9840-9931) | 39 | 4 | 10.3 | 2 | 5.1 |
| Leukaemia NOS (M9800-9801) | 2 | 2 | 100 | 0 | 0.0 |
| Melanoma (M8720-8790) | 19 | 7 | 36.8 | 3 | 15.8 |
| Total | 5436 | 1833 | 33.7 | 6 | 17.1 |
